# Influence of W substitution on crystal structure, phase evolution and microwave dielectric properties of (Na_0.5_Bi_0.5_)MoO_4_ ceramics with low sintering temperature

**DOI:** 10.1038/s41598-017-03620-0

**Published:** 2017-06-09

**Authors:** Li-Xia Pang, Di Zhou, Ze-Ming Qi, Zhen-Xing Yue

**Affiliations:** 1 Micro-optoelectronic Systems Laboratories, Xi’an Technological University, Xi’an, 710032 Shaanxi China; 2 Electronic Materials Research Laboratory, Key Laboratory of the Ministry of Education & International Center for Dielectric Research, Xi’an Jiaotong University, Xi’an, 710049 Shaanxi China; 3 Department of Materials Science and Engineering, University of Sheffield, Sheffield, S1 3JD UK; 4 National Synchrotron Radiation Laboratory, University of Science and Technology of China, Anhui 230029 Hefei, China; 5 State Key Laboratory of New Ceramics and Fine Processing, Department of Materials Science and Engineering, Tsinghua University, Beijing, 100084 PR China

## Abstract

In this work, the (Na_0.5_Bi_0.5_)(Mo_1−x_W_x_)O_4_ (x = 0.0, 0.5 and 1.0) ceramics were prepared via solid state reaction method. All the samples can be well densified at sintering temperature about ~720 °C. Dense and homogeneous microstructure with grain size lying between 2~8 μm can be observed from scanning electron microscopy (SEM). Microwave dielectric permittivity of the (Na_0.5_Bi_0.5_)(Mo_0.5_W_0.5_)O_4_ ceramic was found to be temperature-independent in a wide range between 25~120 °C with a temperature coefficient of frequency (TCF) ~−6 ppm/°C, a permittivity ~28.9, and Qf values 12,000~14,000 GHz. Crystal structure was refined using Rietveld method and lattice parameters are *a* = *b* = 5.281 (5) Å and *c* = 11.550 (6) Å with a space group I 4_1_/a (88). The (Na_0.5_Bi_0.5_)(Mo_1−x_W_x_)O_4_ ceramics might be good candidate for low temperature co-fired ceramics (LTCC) technology.

## Introduction

Microwave dielectric ceramics usually possess large dielectric permittivity (ε_r_, compared with that of polymer), small temperature coefficient of frequency (TCF) and low dielectric loss (high quality factor Qf). In recent forty years, microwave dielectric ceramics have been extensively employed as dielectric resonator (DR), dielectric filter, duplexer, dielectric waveguide, dielectric substrate and etc. in mobile communication, radar and global position system (GPS) and other satellite navigation guiding and positioning systems. High integration and miniaturization of modern microwave devices also benefit from the fast development of microwave dielectric materials^1–3^.

In the past forty years, large amount of microwave dielectrics have been explored, such as (Mg,Ca)TiO_3_ system with ε_r_~20, Ba(Mg,Zn)(Ta,Nb)O_3_ system with ε_r_~30–40, (Zr,Sn)TiO_4_ system with ε_r_~40–45, (Sr,Ca)TiO_3_-(La,Nd)AlO_3_ system with ε_r_~45, and BaO-(Sm,Nd)_2_O_3_-TiO_2_ system with ε_r_ above 70^4–8^. However, the high sintering temperature of traditional microwave dielectric ceramics are not suitable for the fast developing modern fabrication technology, low temperature co-fired ceramics (LTCC) technology^2, 3^. Although many lead-rich microwave dielectric ceramics possess low sintering temperature, their toxicity has limited their wide application^9–13^. In recent ten years, the so-called ultra-low temperature co-fired ceramics (ULTCC) technology has attracted much attention due to their intrinsic low firing temperature below base metals’ melting points, such as Al (~660 °C) and Ag (~961 °C)^14, 15^. The top two biggest ULTCC families are Te-rich and Mo-rich series^14–17^. Considering the environmental friendless and price advantage, Mo-rich ULTCC will play an important role in the high integrated electronic devices. Among them, a big family with scheelite-related structure has attracted much attention. Scheelite structure derive from natural mineral CaWO_4_ and got its name after the discoverer chemist C.W. Scheele from Sweden. The typical formula of scheelite structure is ABO_4_, in which A site can be alkali metal ions, alkaline earth metal ions, some trivalent metal ions, such as Li, Na, Ba, Sr, Ca, Bi etc., with eight coordination number and B site can be trivalent, pentavalent or six valence metal ion, such as Fe, Ga V, Mo, W etc., with four coordination number^18^. Meanwhile, the anion O can also be replaced partially by F or N. Scheelite structure is quite adaptive like perovskite structure in ABO_3_ systems. High concentration as large as 1/3 of defects can be introduced into scheelite structure’s A site, in which the formula can be written as A_1−x_Φ_x_MoO_4_. Defects and substitutions can cause the ordering at A site, such as (K_0.5_Eu_0.5_)MoO_4_ and Bi_2/3_Φ_1/3_MoO_4_^19–24^. As reported in literatures, large difference of ionic radius at B site also results in the ordering of [BO_4_] arrangement at B site, such as Bi(Fe_1/3_Mo_2/3_)O_4_^25^. High adaptability of scheelite structure can provide large amount of data to study its structure-property relation. In 2006 and 2007^26, 27^, Hong *et al*. first reported microwave dielectric properties and sintering behaviors of the scheelite structured (Ca,Sr,Ba)(W,Mo)O_4_ ceramics with sintering temperature below 1100 °C, dielectric permittivity between 8~11, Qf values between 30,000 GHz~90,000 GHz, TCF values between −40~−80 ppm/°C. Large negative TCF values limit their application. Subsequently, a series of A site complex substitutions compositions with scheelite structure were reported to possess positive TCF values in our previous work, such as the (Li_0.5_Bi_0.5_)MoO_4_ (ε_r_ = 44.4, Qf = 3,200 GHz and TCF = +245 ppm/°C), (Na_0.5_Bi_0.5_)MoO_4_ (ε_r_ = 34.4, Qf = 12,300 GHz and TCF = +43 ppm/°C) and (Ag_0.5_Bi_0.5_)MoO_4_ (ε_r_ = 30, Qf = 12,600 GHz and TCF = +57 ppm/°C)^28, 29^. When the B site is occupied by W, the (Li_0.5_Bi_0.5_)WO_4_ and (Ag_0.5_Bi_0.5_)WO_4_ compositions crystallize in wolframite structure^30^ and only the (Na_0.5_Bi_0.5_)WO_4_ still keep scheelite structure^31^. These results offer the opportunity to design temperature stable scheelite structured microwave dielectric ceramics and study the relation between structure and TCF values. Besides the wide range adjustability of permittivity, Qf and TCF values, low sintering temperature below 700 °C is also a highlight for the Mo-based scheelite materials. In the present work, phase evolution, crystal structure, microstructure, microwave dielectric properties and their relation in the (Na_0.5_Bi_0.5_)(Mo_1−x_W_x_)O_4_ (x = 0.0, 0.5 and 1.0) system were studied in detail.

**Figure 1:**
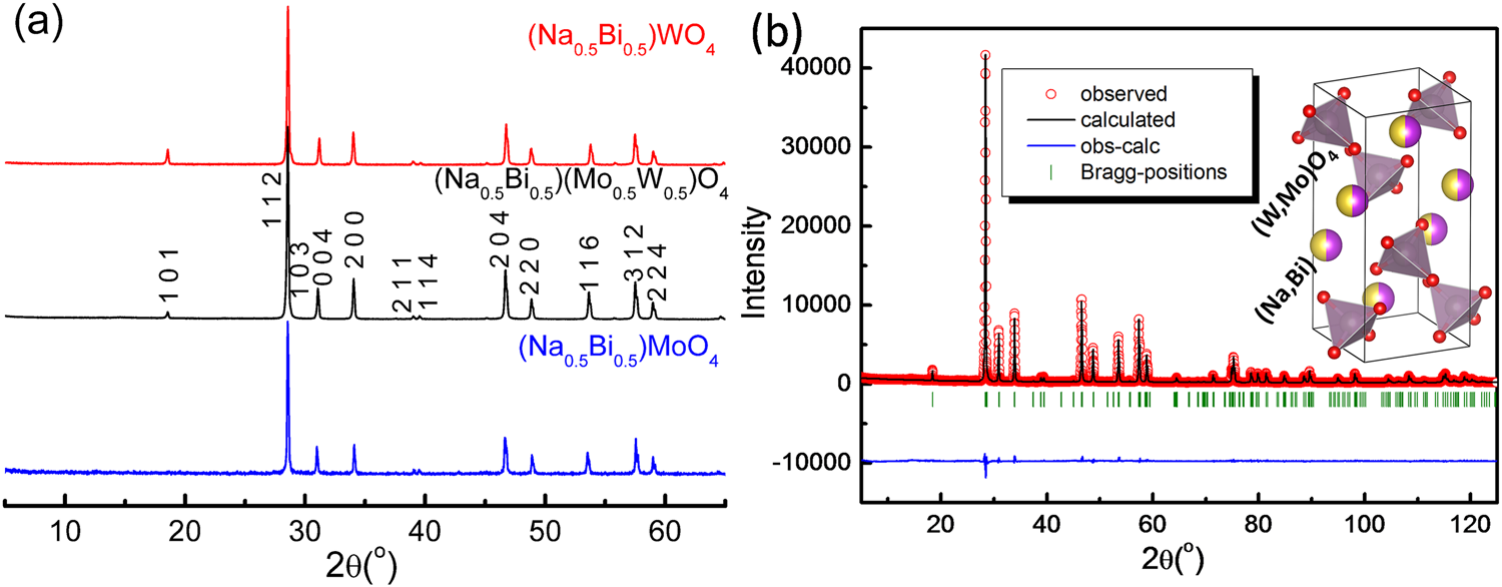
XRD patterns of the (Na_0.5_Bi_0.5_)(Mo_1−x_W_x_)O_4_ (x = 0.0, x = 0.5 and x = 1.0) ceramics (**a**), the experimental (circles) and simulated (line) XRD patterns of the (Na_0.5_Bi_0.5_)(Mo_0.5_W_0.5_)O_4_ composition sintered at 720 °C using space group I 4_1_/a (88) (R_p_ = 9.08%, R_wp_ = 9.95%, R_exp_ = 6.25%) (**b**) (The short vertical lines represent Bragg reflection positions. The continuous line at the bottom shows the difference between experimental and simulated intensity).

XRD patterns of the (Na_0.5_Bi_0.5_)(Mo_1−x_W_x_)O_4_ (x = 0.0, 0.5 and 1.0) ceramics sintered at 720 °C for 2 h are presented in Fig. 1(a). All the samples crystallized in standard tetragonal scheelite structure, in which Na^+^ and Bi^3+^ randomly occupied 8-coordinated A site and Mo^6+^ and W^6+^ randomly occupied 4-coordinated B site. This result is similar to the literatures’ reports^32–34^. This result indicates that tetragonal scheelite structured solid solution was formed in full composition range in the (Na_0.5_Bi_0.5_)(Mo_1−x_W_x_)O_4_ ceramics. To study the crystal structure details of x = 0.5 composition, refinements were performed by using Fullprof software^35^ based on refined XRD data. Measured and calculated XRD patterns are presented in Fig. 1(b). As listed in Table 1, refined cell parameters are *a* = *b* = 5.281 (5) Å and *c* = 11.550 (6) Å. with a space group I4_1_/a (No. 88) and acceptable R_p_ = 9.08%, R_wp_ = 9.95% and R_exp_ = 6.25% using the data (ICSD #67491) reported by Teller as starting model^36^. Cell parameters of the (Na_0.5_Bi_0.5_)MoO_4_ and (Na_0.5_Bi_0.5_) WO_4_ are *a* = *b* = 5.274 (4) Å, *c* = 11.578 (2) Å and *a* = *b* = 5.282 Å, *c* = 11.50 Å, respectively, as reported by Hanuza, Waskowska *et al*.^31, 37, 38^. In scheelite structure, Mo^6+^ is 4-coordinated and its ionic radius is 0.41 Å, while the ionic radius of W^6+^ is 0.60 Å^39^. Hence it is understandable that cell parameters changed almost linearly with W^6+^ substitution concentration.

**Table 1:** Refined atomic fractional coordinates from XRD data of the (Na_0.5_Bi_0.5_)(Mo_0.5_W_0.5_)O_4_ ceramic and the cell parameters are a = b = 5.281 (5) Å, c = 11.550 (6) Å with a space group is I 4_1_/a (88).

Atom	Site	Occ.	x	y	z	Biso
Na	4b	0.125	0.00000	0.25000	0.62500	0.75528
Bi	4b	0.125	0.00000	0.25000	0.62500	0.75528
Mo	4a	0.125	0.00000	0.25000	0.12500	0.60363
W	4a	0.125	0.00000	0.25000	0.12500	0.60363
O	16 f	1.000	0.1491(9)	0.0041(1)	0.2089(5)	0.75169

**Figure 2:**
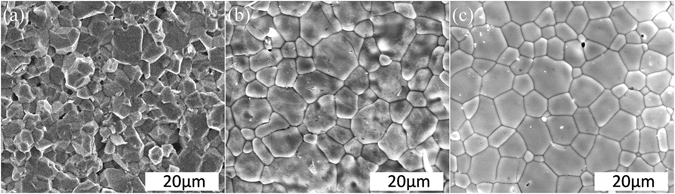
SEM images of the (Na_0.5_Bi_0.5_)(Mo_1−x_W_x_)O_4_ (**a**) x = 0.0 (fractured surface), (**b**) x = 0.5 and (**c**) x = 1.0 ceramics sintered at 720 °C for 2 hr.

Microstructures of the (Na_0.5_Bi_0.5_)(Mo_1−x_W_x_)O_4_ (x = 0.0, 0.5 and 1.0) ceramics sintered at 720 °C for 2 h are shown in Fig. 2. Dense and homogeneous microstructure can be observed, which means that all the ceramics could be well densified at a low sintering temperature ~720 °C. There is no apparent change trend of grains versus substitution of W and it lied between 2~8 µm.

**Figure 3:**
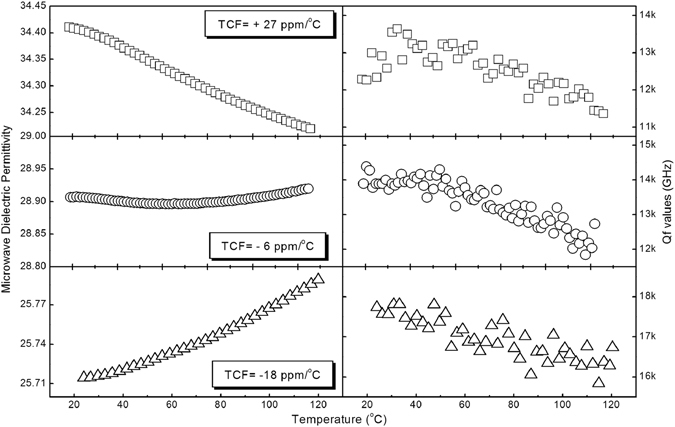
Microwave dielectric permittivities and Qf values of the (Na_0.5_Bi_0.5_)(Mo_1−x_W_x_)O_4_ (x = 0, 0.5, 1.0) ceramics as a function of temperature.

Phase compositions, sintering temperatures and microwave frequency dielectric properties of the (A_0.5_Bi_0.5_)(Mo,W)O_4_ (A = Li, Na and Ag) ceramics were listed in Table 2 ^28–30, 40, 41^. The microwave dielectric properties of (Na_0.5_Bi_0.5_)WO_4_ were first reported here with a ɛ_r_~25.7, a Qf value ~17,500 GHz and a τ_f_ value ~− 18 ppm/°C. Although the permittivity of (Na_0.5_Bi_0.5_)WO_4_ ceramic is a little smaller than that of (Na_0.5_Bi_0.5_)MoO_4_ ceramic, its Qf value is 1.4 times larger than that of the (Na_0.5_Bi_0.5_)MoO_4_ ceramic. It must be noted that their TCF values are opposite and this provides an opportunity to design a temperature stable (Na_0.5_Bi_0.5_)(Mo_0.5_W_0.5_)O_4_ solid solution ceramic with a ɛ_r_~28.9, a Qf value ~14,000 GHz and a τ_f_ value ~−6 ppm/°C as listed in Table 2. As mentioned above, full solid solution can be formed in the (Na_0.5_Bi_0.5_)(Mo_1−x_W_x_)O_4_ ceramics. It is quite different from the situation in (Li_0.5_Bi_0.5_)(Mo_1−x_W_x_)O_4_ system, in which composite samples were formed. Besides, it is also quite different from the situation in (Ag_0.5_Bi_0.5_)(Mo_1−x_W_x_)O_4_ system, in which limited solid solution were formed. Compared with composite method, solid solution method is more controllable. Change of permittivity might be explained by Shannon’s additive rule^42^. According to Shannon’s results, the polarizabilities *α*_*x*_ of (Na_0.5_Bi_0.5_)(Mo_1−x_W_x_)O_4_ could be calculated as follows:

1 $${\alpha }_{x}=0.5\times ({\alpha }_{B{i}^{3+}}+{\alpha }_{N{a}^{+}})+x{\alpha }_{{W}^{6+}}+(1-x){\alpha }_{M{o}^{6+}}+4{\alpha }_{{O}^{2-}}$$

where ${\alpha }_{B{i}^{3+}}$, ${\alpha }_{{O}^{2-}}$, ${\alpha }_{M{o}^{6+}}$, ${\alpha }_{N{a}^{+}}$ and ${\alpha }_{{W}^{6+}}$ are the polarizabilities of Bi^3+^, O^2−^, Mo^6+^, Na^+^ and W^6+^, respectively^26, 27, 42^. Using the Clausius-Mossotti relation, the relation between dielectric permittivity ε_r_, polarizability α_x_, cell volume V_x_, and the dielectric constant are obtained as following:

2 $${\varepsilon }_{x}=\frac{3{V}_{x}+8\pi {\alpha }_{x}}{3{V}_{x}-4\pi {\alpha }_{x}}\iff {\alpha }_{x}=\frac{3{V}_{x}}{4\pi }\frac{{\varepsilon }_{x}-1}{{\varepsilon }_{x}+2}$$

in which V_x_ is cell volume and ε_x_ is the permittivity of (Na_0.5_Bi_0.5_)(Mo_1−x_W_x_)O_4_ ceramics. The calculated molecular polarzabilites from equations (1) and (2) and macroscopical microwave dielectric properties were listed in Table 3. Because the ionic polarizability of Mo^6+^ is larger than that of W^6+^, it is understandable that permittivity decreased with increase of W content. The relative error of molecular polarzability is below 13%, which is acceptable and might be attributed to the specific ion neighboring environment. To better understand the scheelite solid solubility here, the phase compositions, sintering temperatures and microwave dielectric properties of the (A_0.5_Bi_0.5_)(Mo,W)O_4_ (A = Li, Na, K and Ag) ceramics are presented in Table 2 in order of ionic radius of A^+^. It is seen that the scheelite solid solubility is influenced both by the equivalent ionic radius on A and B sites. According to our previous work, scheelite solid solubility is less than 40% in the (Li_0.5_Bi_0.5_)(Mo_1−x_W_x_)O_4_ system and possibly larger than 50% in the (Ag_0.5_Bi_0.5_)(Mo_1−x_W_x_)O_4_ system, while scheelite solid solution was formed in the whole composition range in (Na_0.5_Bi_0.5_)(Mo_1−x_W_x_)O_4_ system here. According to Errandonea and Manjon’ review ref. 43, structures of ABX_4_ materials are determined both by the ionic radius of A, B, and X ions and the pressure. It is not easy to give a simple structural factor for scheelite structure, such as the famous tolerance factor defined as (R_A_ + R_O_)/(R_B_ + R_O_) in perovskite structure^44^. Anyway, from the experimental data listed in Table 2, we can only give a conclusion that scheelite structure can only be kept within a limited range of radius of ions on both A and B sites in oxides. The microwave dielectric permittivities and Qf values of (Na_0.5_Bi_0.5_)(Mo_1−x_W_x_)O_4_ (x = 0, 0.5, 1.0) ceramics as a function of temperature are shown in Fig. 3. It is clearly seen that substitution of W for M in the (Na_0.5_Bi_0.5_)MoO_4_ ceramic efficiently modify the temperature dependence of permittivity. In a wide temperature range 20~120 °C, the permittivity of (Na_0.5_Bi_0.5_)(Mo_0.5_W_0.5_)O_4_ ceramic changed slightly around 28.9, while Qf values above 12,000 GHz. It is interesting to note that no matter in forms of solid solution or composite, TCF values can be easily tailored to be near zero in the (A_0.5_Bi_0.5_)(Mo,W)O_4_ (A = Li, Na, K and Ag) systems, which makes them possible in fabrication of temperature stable devices. Infrared reflectivity fitting was also employed to study the intrinsic dielectric properties and details were presented in Supplemental Information. Bi^3+^ neighboring structure was found to account for the shift of macroscopical permittivity due to the large polarization ~6.12 Å^3^, which is larger than that of Mo^6+^ and W^6+^ ions (3.28 and 3.2 Å^3^, respectively)^26, 27, 42^.

**Table 2:** Phase compositions, sintering temperatures (S. T.) and Microwave dielectric properties of the (A_0.5_Bi_0.5_)(Mo,W)O_4_ (A = Li, Na, K and Ag) ceramics.

Composition	Phase	S.T. (°C)	ε_r_	Qf (GHz)	TCF (ppm/°C)	A site radius	B site Radius	Refs
(Li_0.5_Bi_0.5_)MoO_4_	scheelite	560	44.4	3,200	+245	1.045	0.41	28
(Li_0.5_Bi_0.5_)(Mo_0.4_W_0.6_)O_4_	composite	620	31.5	8,500	+20	1.045	0.416	30
(Li_0.5_Bi_0.5_) WO_4_	wolframite	740	27.2	17,000	−56	1.045	0.42	30
(Na_0.5_Bi_0.5_)MoO_4_	scheelite	690	34.4	12,300	+43	1.175	0.41	29
(Na_0.5_Bi_0.5_)(Mo_0.5_W_0.5_)O_4_	scheelite	720	28.9	14,000	−6	1.175	0.415	this work
(Na_0.5_Bi_0.5_) WO_4_	scheelite	720	25.7	17,500	−18	1.175	0.42	this work
(Ag_0.5_Bi_0.5_)MoO_4_	scheelite	690	30.4	12,600	+57	1.225	0.41	29
(Ag_0.5_Bi_0.5_)(Mo_0.5_W_0.5_)O_4_	scheelite	580	26.3	10,000	+20	1.225	0.415	40
(Ag_0.5_Bi_0.5_)WO_4_	wolframite	580	35.9	13,000	−69	1.225	0.42	41
(K_0.5_Bi_0.5_)MoO_4_	monoclinic	630	37	4,000	+117	1.34	0.41	29

**Table 3:** Dielectric permittivity, cell volume, calculated and measured molecular polarizability of the (Na_0.5_Bi_0.5_)(Mo_1−x_W_x_)O_4_ ceramics.

Composition	ε_r_	Cell volume (Å^3^)	α_meas_ (Å^3^)	α_cal_ (Å^3^)	Ref.
(Na_0.5_Bi_0.5_)MoO_4_	34.4	322.09/4	17.639	15.28	17
(Na_0.5_Bi_0.5_)(Mo_0.5_W_0.5_)O_4_	28.9	322.20/4	17.363	15.24	this work
(Na_0.5_Bi_0.5_)WO_4_	25.7	319.80/4	17.019	15.2	this work

## Summary

Different from the (Li_0.5_Bi_0.5_)(Mo,W)O_4_ and (Ag_0.5_Bi_0.5_)(Mo,W)O_4_ system, with a ionic radius of 1.18 Å (Na^+^) between that of Li^+^ and Ag^+^, (Na_0.5_Bi_0.5_)(Mo,W)O_4_ system can easily crystallize in standard scheelite structure. Crystal structure refinement based on Rietveld method gave the cell parameters a = b = 5.281 (5) Å, and c = 11.550 (6) Å with a space group I 4_1_/a (88). With increase of W content, microwave permittivity decreased from 34.4 to 25.7 and Qf value increased from 12,300 GHz to 17,500 GHz while TCF shifting from +43 to −18 ppm/°C, which indicates that TCF value is strongly dependent on the [BO_4_] tetrahedron in scheelite structure. Far-infrared reflectivity further confirmed that phonons at infrared region contributed main dielectric polarization at microwave region. The (Na_0.5_Bi_0.5_)(Mo,W)O_4_ system could be good candidate for Pb-free ULTCC technology.

## Methods

### summary

The (Na_0.5_Bi_0.5_)(Mo_1−x_W_x_)O_4_ (x = 0.0, 0.5 and 1.0) ceramics were prepared via the traditional solid state reaction method as described in our previous work^2, 15^. The calcination temperature is 600 °C and the samples were sintered under air atmosphere in 700~740 °C. XRD was performed using a Rigaku D/MAX-2400 X-ray diffractometry with Cu Kα radiation. The Rietveld profile refinement method was performed on chosen resutls, using Fullprof program^35^. Ceramic surfaces were examined by a FEI Quanta 250 F scanning electron microscopy (SEM). Infrared reflectivity spectra were collected using a Bruker IFS 66 v FTIR spectrometer (on Infrared beamline station (BL01B) at National Synchrotron Radiation Laboratory (NSRL), China). Microwave frequency dielectric properties were obtained using the TE_01δ_ dielectric resonator method^45^ with a HP 8720 Network Analyzer and a thermal cycling chamber (Delta 9023, Delta Design, Poway, CA). The temperature coefficient of frequency TCF (τ_*f*_) was obtained using the following formula:

3 $$TCF({\tau }_{f})=\frac{{f}_{T}-{f}_{{T}_{0}}}{{f}_{{T}_{0}}\times (T-{T}_{0})}\times {10}^{6}$$

where the *f*_T_ and *f*_T0_ were the TE_01δ_ frequencies at temperature T and T_0_, respectively.

## Electronic supplementary material

supplemental information
