# Supplementary material for: Influence of W substitution on crystal structure, phase evolution and microwave dielectric properties of (Na0.5Bi0.5)MoO4 ceramics with low sintering temperature
Source: Sci Rep. 2017 Jun 9;7:3201. doi: 10.1038/s41598-017-03620-0 (PMC5466633; doi:10.1038/s41598-017-03620-0)
Supplement: Supplementary file 1 — supplemental information [file 41598_2017_3620_MOESM1_ESM.doc]

# Influence of W substitution on crystal structure, phase evolution and microwave dielectric properties of (Na_0.5_Bi_0.5_)MoO_4_ ceramics with low sintering temperature

Li-Xia Pang,^1^ Di Zhou,[[1]](#footnote-2)*2,3 Ze-Ming Qi,^4^ and Zhen-Xing Yue^5^

^1^Micro-optoelectronic Systems Laboratories, Xi’an Technological University, Xi’an 710032, Shaanxi, China

^2^Electronic Materials Research Laboratory, Key Laboratory of the Ministry of Education & International Center for Dielectric Research, Xi’an Jiaotong University, Xi’an 710049, Shaanxi, China

^3^Department of Materials Science and Engineering, University of Sheffield, S1 3JD, UK

^4^National Synchrotron Radiation Laboratory, University of Science and Technology of China, Anhui, 230029, Hefei, China

^5^State Key Laboratory of New Ceramics and Fine Processing, Department of Materials Science and Engineering, Tsinghua University, Beijing 100084, PR China

Infrared Reflectivity Fitting Analysis

To better understand the intrinsic dielectric properties, considering the Fresnel relation between infrared reflectivity and complex permittivity, far-infrared reflectivity spectra of the (Na_0.5_Bi_0.5_)(Mo_1-x_W_x_)O_4_ (x=0, 0.5, 1.0) ceramics were fitted using Lorentz three-parameter classical oscillator model as following:

$R=\left|\frac{\sqrt{\varepsilon^{*}(\omega)}-1}{\sqrt{\varepsilon^{*}(\omega)}+1}\right|^{2}$ (1)

$\varepsilon^{*}(\omega)=\varepsilon_{\infty}+\sum_{j=1}^{n} \frac{\omega_{jTO}^{2}S_{jTO}}{\omega_{jTO}^{2}-\omega^{2}+i\omega\gamma_{jTO}}$ (2)

where R is the infrared reflectivity; ɛ*(ω) is the complex permittivity; *ε_∞_* is optical frequency dielectric constant caused by electronic polarization; *n* is the number of infrared modes; *ω_j_*_TO_ and *ω_j_*_LO_ are frequencies of the *j*^th^ transverse optical mode and the *j*^th^ longitudinal optical mode; *S_j_*_TO_ is the strength of the *j*^th^ transverse optical mode. The fitting results are presented in Fig. S1. It is seen that there are only small differences observed in reflectivity in wave-number range 200 ~ 450 cm^-1^, which can be assigned to the stretching of [BO_4_] tetrahedron. It can be deduced that the TCF values, which is mainly determined by temperature dependence of permittivity, might be strongly related with the stretching of both [WO_4_] and [MoO_4_] tetrahedrons here. However, we do not have *in-situ* infrared reflectivity data to study the details. Fig. S1b presents both the measured and fitted values of real and imaginary parts of permittivity. It can be seen that both the measured and extrapolated values corresponded well with each other, which confirms that main dielectric polarization at microwave region arise from the phonon at infrared region. The phonon parameters obtained from the fitting of the infrared reflectivity spectra of the (Na_0.5_Bi_0.5_)(Mo_0.5_W_0.5_)O_4_ and (Na_0.5_Bi_0.5_)WO_4_ ceramic are listed in Table S1 and S2. As seen from the phonon parameters listed in the tables below, the dielectric polarization contribution from the vibration modes below 150 cm^-1^ (No. 1 and No. 2 modes) can reach above 75 % of the total value for x=0.5 and 1 samples. This result is quite different from the situations for low permittivity materials, such as Ag_2_MoO_4_, NaAgMoO_4_, etc. This implies that the dielectric polarization contribution of Bi-based scheelite solid solution mainly comes from the external modes, which means the vibrational modes of Bi-O stretches. It is understandable that the neighboring structure of Bi^3+^ can account for the change of macroscopical permittivity due to its large polarization about 6.12 Å^3^, which is much larger than that of Mo^6+^ and W^6+^ ions (3.28 and 3.2 Å^3^, respectively).


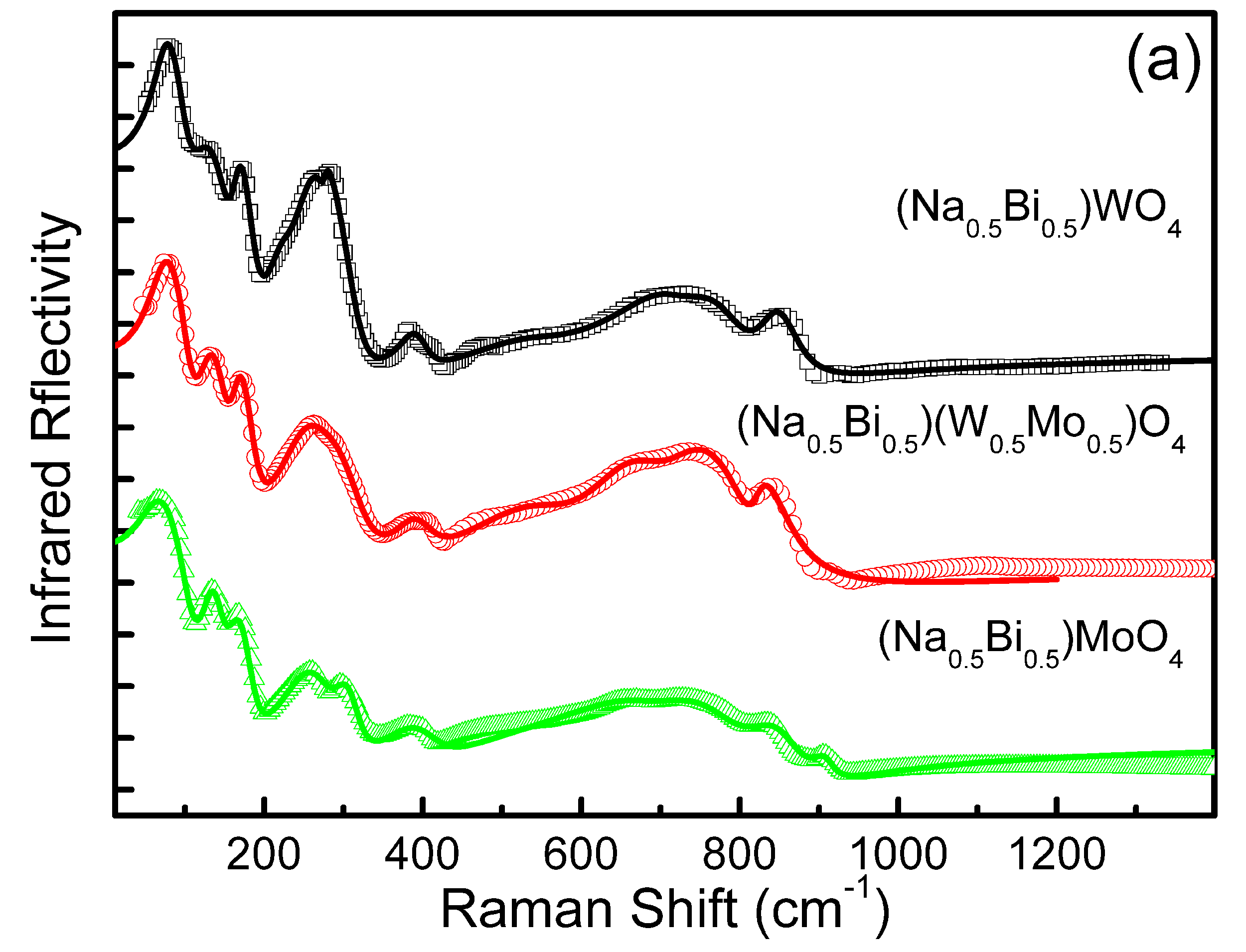

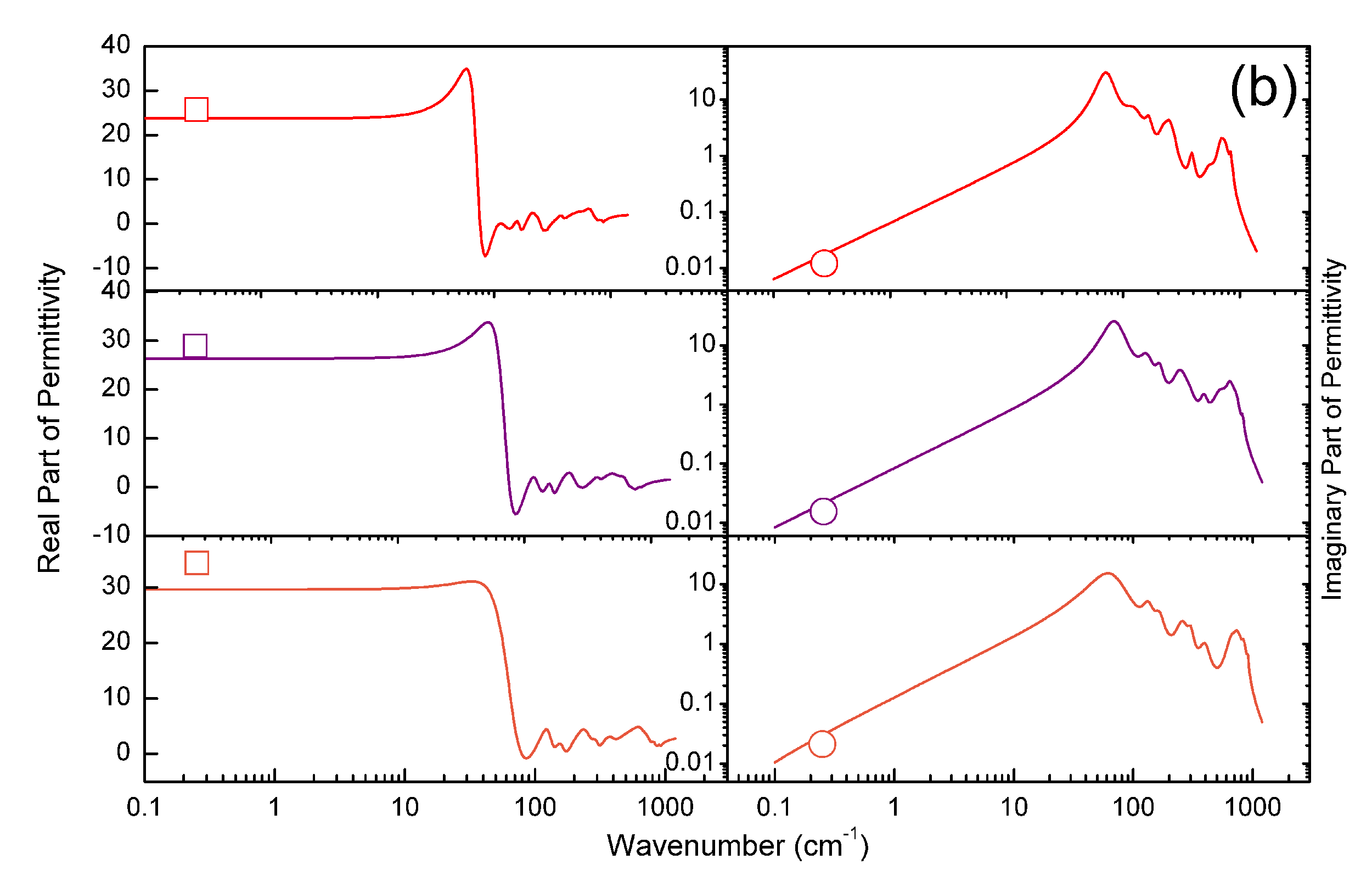


Fig. S1. Measured and calculated infrared reflectivity spectra (a) (solid line for fitting values and circle for measured values) and fitted complex dielectric spectra of the (Na_0.5_Bi_0.5_)(Mo_1-x_W_x_)O_4_ (x=0, 0.5, 1.0) ceramics (b) (circles are experimental at microwave region)

Table S1. Phonon parameters obtained from the fitting of the infrared reflectivity spectra of the (Na_0.5_Bi_0.5_)(Mo_0.5_W_0.5_)O_4_ ceramic

| Mode | *ω_oj_* | *ω_pj_* | *γ_j_* | Δ***ε****_j_* |
| --- | --- | --- | --- | --- |
| 1 | 70.958 | 302.84 | 33.761 | 18.2 |
| 2 | 129.17 | 196.14 | 38.6 | 2.31 |
| 3 | 165.52 | 135.61 | 25.937 | 0.671 |
| 4 | 248.03 | 288.23 | 72.549 | 1.35 |
| 5 | 286.68 | 162.64 | 79.882 | 0.322 |
| 6 | 390.27 | 167.05 | 59.717 | 0.183 |
| 7 | 536.63 | 413.01 | 164.29 | 0.592 |
| 8 | 646.58 | 459.76 | 119.4 | 0.506 |
| 9 | 724.43 | 294.63 | 107.38 | 0.165 |
| 10 | 821.24 | 106.54 | 36.083 | 0.0168 |
| x=0.5 | *ε*_∞_=2.02 | | *ε*_0_=26.35 | |

Table S2. Phonon parameters obtained from the fitting of the infrared reflectivity spectra of the (Na_0.5_Bi_0.5_)WO_4_ ceramic

| Mode | *ω_oj_* | *ω_pj_* | *γ_j_* | Δ***ε****_j_* |
| --- | --- | --- | --- | --- |
| 1 | 72.037 | 279.94 | 26.09 | 15.1 |
| 2 | 124.64 | 233.49 | 56.977 | 3.51 |
| 3 | 165.19 | 122.46 | 23.021 | 0.55 |
| 4 | 220.77 | 127.98 | 37.7 | 0.336 |
| 5 | 249.62 | 250.79 | 50.592 | 1.01 |
| 6 | 276.13 | 39.158 | 11.585 | 0.02 |
| 7 | 389.06 | 138.93 | 42.778 | 0.128 |
| 8 | 548.53 | 199.7 | 138.63 | 0.133 |
| 9 | 695.93 | 421.07 | 111.56 | 0.366 |
| 10 | 756.21 | 316.19 | 100.82 | 0.175 |
| 11 | 838.03 | 206.11 | 50.346 | 0.0605 |
| x=1 | *ε*_∞_=2.37 | | *ε*_0_=23.76 | |

1. ^^Corresponding author. Tel.: +86-29-82668679; E-mail address: zhoudi1220@gmail.com (Di Zhou) [↑](#footnote-ref-2)
